# Comparison of Two Methods – Regression Predictive Model and Intake Shift Model – For Adjusting Self-Reported Dietary Recall of Total Energy Intake of Populations

**DOI:** 10.3389/fpubh.2014.00249

**Published:** 2014-11-27

**Authors:** Joanna Lankester, Sharon Perry, Julie Parsonnet

**Affiliations:** ^1^Department of Electrical Engineering, Stanford University, Stanford, CA, USA; ^2^Department of Medicine, Stanford University School of Medicine, Stanford, CA, USA; ^3^Department of Health Research and Policy, Stanford University School of Medicine, Stanford, CA, USA

**Keywords:** bias (epidemiology), computer simulation, diet surveys, energy intake, NHANES, questionnaires

## Abstract

Daily dietary intake data derived from self-reported dietary recall surveys are widely considered inaccurate. In this study, methods were developed for adjusting these dietary recalls to more plausible values. In a simulation model of two National Health and Nutrition Examination Surveys (NHANES), NHANES I and NHANES 2007–2008, a predicted one-third of raw data fell outside a range of physiologically plausible bounds for dietary intake (designated a 33% failure rate baseline). To explore the nature and magnitude of this bias, primary data obtained from an observational study were used to derive models that predicted more plausible dietary intake. Two models were then applied for correcting dietary recall bias in the NHANES datasets: (a) a linear regression to model percent under-reporting as a function of subject characteristics and (b) a shift of dietary intake reports to align with experimental data on energy expenditure. After adjustment, the failure rates improved to <2% with the regression model and 4–9% with the intake shift model – both substantial improvements over the raw data. Both methods gave more reliable estimates of plausible dietary intake based on dietary recall and have the potential for more far-reaching application in correction of self-reported exposures.

## Introduction

Research on diet and obesity has long been hampered by inaccurate estimates of how much we, as individuals and as a population, eat. Individual daily food consumption is often gathered through 24-h dietary recalls, in which individuals report foods and quantities consumed in a given day and total energy intake (EI) is derived.

Dietary recall bias is a well-studied problem in nutrition research (1–6). In general, individuals tend to under-report their food consumption (1, 3, 7, 8). Substantial efforts are made to minimize biases in primary data collection. For example, dietary surveys such as National Health and Nutrition Examination Survey (NHANES) use visual aids to help participants recall food quantities, and guidelines detail how to ask questions in a neutral manner (9). Despite these efforts, individuals continue to misreport.

Some error also arises from the assumption that dietary recalls inform the average EI value, while in reality there is day-to-day variation within individuals so that a dietary recall from a particular day could indicate an intake above or below the value researchers are trying to measure. To adjust for this variation – i.e., to improve precision – researchers have applied a variety of corrections (10) such as the NRC (11), NRC-B (4), and Iowa State University methods (12, 13). Models have also been developed to quantify relationships between EI and intake of particular nutrients (14, 15) or to infer EI from biomarkers in urine (16). Still, no method exists to improve bias of the distribution of total EI from dietary recalls in a population using easily measured characteristics of survey participants.

To gain better insight from dietary recall data, especially in epidemiological nutrition models, a method of correcting for misreporting of total EI in a population would be beneficial. Using an observational dataset from the Observing Protein and Energy Nutrition (OPEN) study, we developed two methods to predict corrected dietary recall data. We then tested these methods on NHANES I (17) and NHANES 2007–2008 (18) datasets by adjusting dietary recall for a population sample. We chose NHANES because of its thorough survey methodology in capturing population health data and its collection over several decades of time, and we chose these particular NHANES datasets as the most and least recent datasets at the time of this analysis.

## Materials and Methods

**Figure 1:**
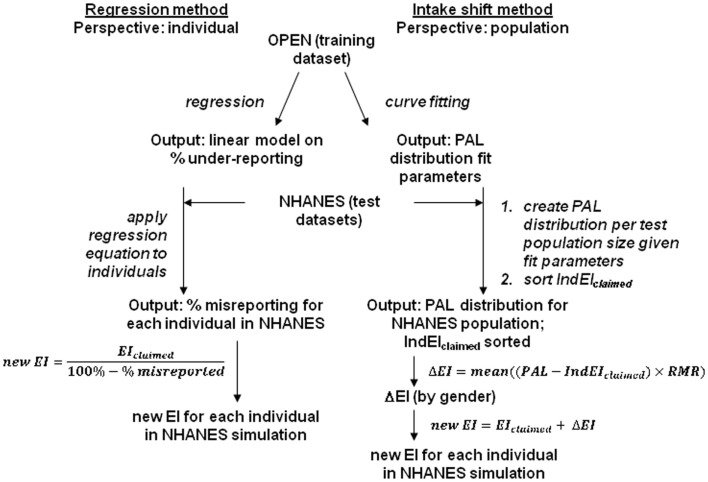
**Flow chart for the development and implementation of dietary recall correction models**. Left side, regression model: right side, intake shift model. Two separate analyses on the OPEN dataset produced output models that were then used in simulation models with National Health and Nutrition Examination Survey (NHANES) datasets. The simulations gave final outputs that were used to estimate a corrected energy intake (EI). Physical activity level (PAL) = energy expenditure/resting metabolic rate (RMR); IndEI_claimed_ = EI_claimed_/RMR; OPEN = observing protein and energy nutrition.

We used a dataset from the OPEN study to develop a model for under-reporting and used two different NHANES datasets to test the model (Figure 1).

### Training dataset (OPEN study) and test datasets (NHANES)

We requested the dataset from the OPEN study author (19). The OPEN study compared self-reported caloric dietary intake with measurements of energy expenditure (EE) in 484 participants ages 40–69 years old. Exclusion criteria for the study included those on a weight loss diet. Subjects self-reported 24-h dietary intake. The investigators then measured their EE using doubly labeled water.

In our analysis, we excluded OPEN subjects who did not complete the doubly labeled water test, leaving 451 individuals in the dataset. Of those remaining, participants were predominantly White non-Hispanic (82% of the participants). Because initial analysis noted a significant difference in reporting between Blacks and non-Blacks, we excluded Blacks from the modeling analysis, leaving a total of 423 subjects.

The publicly available NHANES datasets were downloaded from the Centers for Disease Control and Prevention NHANES website; survey method descriptions are also available (20). In brief, variables included in our analysis were collected through either anthropometric measurements or self-reported survey. The dietary survey included a listing of all foods consumed within a 24-h period and an estimate of their quantities.

We excluded individuals from the NHANES datasets who reported that they were on a special diet or did not eat their typical diet that day, as well as those who did not report at all. We constrained the minimum age to 18; maximum age recorded was 74 in NHANES I; and 79 in NHANES 2007–2008. After these exclusions, the dataset sizes were 8,006 individuals for NHANES I and 4,830 for NHANES 2007–2008.

For both datasets, univariate analyses included means and standard deviations for continuous variables and percentiles for categorical variables as appropriate.

#### Parameters created

We used reported dietary intake information for each eligible OPEN record along with the estimated EE to estimate the accuracy of dietary recall, using the principle that in energy balance, EE should approximate dietary intake.

Variables defined for this analysis included:

1. Resting metabolic rate (RMR). We calculated theoretical RMR for individuals using the Schofield equations (21).
2. Energy expenditure and EI. In energy homeostasis, EE is expected to equal EI (thus, body weight is not changing). The term EI_claimed_ denotes self-reported EI in the OPEN dataset, while the term EI refers to the true EI that the models later estimated.
3. Physical activity level (PAL). PAL is the ratio of EE/RMR. EE cannot biologically be less than RMR, so PAL cannot be <1.
4. Index of energy intake (IndEI). IndEI is the ratio of EI/RMR. In energy homeostasis, EI = EE, so IndEI will equal PAL and will then also have a lower bound of 1. IndEI_claimed_ denotes IndEI measures derived from self-reported EI in the OPEN dataset, i.e., IndEI_claimed_ = EI_claimed_/RMR. This definition allows comparison of EI_claimed_ between two individuals with drastically different RMR and therefore different EI needs. It also allows comparison between IndEI_claimed_ from the OPEN study and figures for PAL from other sources.
5. Percent misreporting. Because subjects reported eating normally, we assumed homeostasis (EI = EE) and defined percent under-reporting as (EE − EI_claimed_)/EE.

### Statistical modeling algorithms

We developed several methods for adjusting a distribution of dietary recall to correct for both under- and over-reporting. All methods assumed energy homeostasis, i.e., body weight is not changing so that EE equal to EI. These included: (a) a linear regression, (b) the shifting of the population’s reported intake by an added average caloric offset, (c) the scaling of population’s intake by a multiplicative value, and (d) the random selection of a dietary intake bias for each individual. The two models, which yielded biologically plausible results in terms of ratio to RMR – (a) and (b) – are presented here. We used SAS 9.2 (SAS Institute, Cary, NC, USA) for statistical analyses.

#### Estimation of individual misreporting error (OPEN indicators regression method)

We estimated using a multiple linear regression on the OPEN data the bias in reported EI based on individuals’ characteristics and self-reported data. The outcome for this model was percent energy misreporting [i.e., (EE − EI_claimed_)/EE, where positive values represent under-reporting and negative values represent over-reporting]. We created a quantile–quantile plot of percent energy misreporting and found that this variable was generally normally distributed, with individuals slightly deviating at the most extreme over-reporting end. Because this represents only a small amount of data, particularly because most individuals under-report, we were satisfied that the normality of the data was sufficient for a linear model. Parameter estimates from this model allowed later prediction of true dietary intake. This method relies on the assumption that misreporting patterns in the OPEN study were similar to those in test datasets.

Predictors considered for the regression model included age, sex, weight, height, body mass index (BMI), Ponderal Index, EI_claimed_, log(EI_claimed_), IndEI_claimed_, and log(IndEI_claimed_). We considered day of the week by checking for trends in mean percent misreporting for each day. We also examined interaction effects for race and ethnicity. A stepwise backwards test was used to eliminate each variable with the highest two-sided *P*-value above a cut-off of 0.05 and to identify collinear terms. We checked residual plots for each variable in the final model for reasonable homogeneity of variance.

#### Estimation of population-level energy intake bias (intake shift method)

The intake shift method’s outcome is the population-average caloric shift in EI, stratified by gender, which should be applied to each individual in a test dataset in order to align the self-reported EI_claimed_ with the measured EE from the OPEN dataset, normalizing both for RMR. This method relies on the assumption that the EE distribution in the OPEN dataset was similar to that of test datasets.

We first analyzed the sex and weight profile distributions of PAL in the OPEN study. Weight status categories were defined using CDC and WHO definitions: BMI <18.5 underweight, >30 obese, >25 overweight, and otherwise normal weight (22, 23). As a result, PAL was subsequently stratified by gender and not by weight status.

To find the shift in EI, we built a simulation with *n* individuals and interpolated the OPEN PAL distribution to *n* individuals. To do this, we first fit the PAL distributions for men and women in OPEN to a log-normal curve. A new curve was created with the mean and standard deviation from that fit and the number of individuals in the simulation of the corresponding sex. After ranking the PAL and the IndEI_claimed_ values, this interpolation allows, for example, the 28th woman in the simulation of *n* individuals to correspond to the would-be placement of the 28th woman in the OPEN data if the OPEN data had *n* individuals. The difference between PAL and IndEI_claimed_ and corresponding change in EI was then calculated for each person in the simulation. These ΔEI were averaged to find a number of calories by which the EI_claimed_ of each person in the simulation should be shifted.

#### Simulation of application of models to NHANES data

We applied these models to simulations of two test datasets, NHANES I and NHANES 2007–2008. Individuals were initialized with the age–sex distribution corresponding to the census population distribution for the year of each dataset. For each individual, characteristics of height, weight, and calories from the dietary recall (EI_claimed_) were drawn randomly from the corresponding age–sex combination distribution in the NHANES test dataset.

Both correction models were run on simulations of three different populations from the NHANES I and NHANES 2007–2008 populations: all adults in the population, non-Blacks only, and non-Blacks age 40–69 only (i.e., same as those in the OPEN study), in order to analyze whether the age and race limitations of the OPEN dataset affected the final outcome.

We ran 1,000 simulations of 10,000 individuals each for each dataset-population-adjustment method combination (12 combinations). A “failure rate” designates the average from all 1,000 simulations of percent of individuals with a PAL out of bounds (i.e., <1 or > maximum value for gender). Simulations were built in MATLAB (Mathworks, Natick, MA, USA).

#### Definition of boundary values for estimating accuracy of self-reported dietary intake

By definition PAL can be no lower than 1, and the Goldberg equations – which are based on measured RMR – establish an even higher lower limit of normal PAL at 1.35 (24). However, Goldberg tabulates data with a PAL as low as 1.16 from a whole-body calorimetry study and acknowledges some variation in the accuracy of calculation of RMR for a given individual. In addition, in the OPEN study the lowest PAL derived from observed EE data was 1.2. Given these results, we used a minimum PAL, and thus a minimum IndEI, of 1. Experimentally derived PAL values from a variety of studies, including among professional athletes, are tabulated in Black et al. (25). In the application of these methods to the general US population, we chose cut-offs of maximum PAL = IndEI_claimed_ as 2.8 for women and 3.5 for men as numbers corresponding to individuals who were extremely active yet were not undertaking long athletic feats such as Arctic exploration or the Tour de France. Values of IndEI_claimed_ outside the specified range (<1.0 and >2.8 for women or 3.5 for men) represent IndEI values that are not physiologically plausible in energy homeostasis, so individuals with these values are considered to have misreported dietary intake.

## Results

Univariate summaries of each dataset are presented in Table 1.

**Table 1:** **Characteristics of individuals in OPEN and NHANES datasets**.

Variable	Mean (standard deviation) or count (percentage), OPEN	Mean (standard deviation) or count (percentage), NHANES I	Mean (standard deviation) or count (percentage), NHANES 2007–2008
Count	451	8006	4830
Age (years)	53.5 (8.4)	47.0 (17.9)	48.4 (8.4)
Female	206 (45.7)	4680 (58.4)	2358 (48.8)
Race
White non-Hispanic	371 (82.3)	6736 (84.1)	2234 (46.3)
Black non-Hispanic	28 (6.2)	1169 (14.6)	1020 (21.1)
Hispanic, any race	18 (4.0)	^a^	1387 (28.7)
Other/unspecified	34 (7.5)	101 (1.3)	189 (3.9)
Weight (kg)	81.0 (17.6)	69.2 (15.3)	80.0 (20.7)
Body mass index (kg/m^2^)	27.8 (5.2)	24.9 (4.8)	28.4 (6.5)
EI_claimed_ (kcal)	2346 (808)	1876 (884)	2120 (1050)
TEE (kcal)	2627 (556)	n/a	n/a

*^a^NHANES I did not track ethnicity (Hispanic vs. non-Hispanic); numbers reflect categories White, Black, and Other*.

### Final OPEN indicators regression model of percent misreporting

In the regression model for misreporting using the OPEN data, younger age, greater weight, male sex, and lower EI claimed, both absolute and adjusted for RMR [i.e., log (EI_claimed_) and IndEI_claimed_] were significantly linked to higher dietary under-reporting (Table 2, all *P*-values two-sided). We found no significant interaction among these terms and no systematic variation in mean under-reporting by day of the week of the dietary recall.

**Table 2:** **Parameter estimates for non-Blacks in the OPEN study for outcome variable % misreporting^a^**.

Variable	Parameter estimate
Intercept	298.19***
Sex (1 = male, 2 = female)	−2.30^a^
Age (years)	−0.36***
Weight (kg)	0.21***
log (EI_claimed_) (log kcal)	−35.84***
IndEI_claimed_ (unitless)	−30.47 ∗∗∗

***P < 0.001,

*^a^NS. *R*^2^ = 0.84. EI_claimed_ = energy intake according to dietary recall, IndEI_claimed_ = EI_claimed_/resting metabolic rate. Positive outcome represents under-reporting*.

### Final intake shift model

The derived PAL distributions were found to differ significantly by gender (*P* < 0.001, males mean 1.83 (SD 1.14); females mean 1.63 (SD 1.13)]. Consistent with other studies (26), the PAL did not differ by weight status (underweight, normal, overweight, and obese) (*P* = 0.69). The PAL distributions for each gender were then used to adjust IndEI_claimed_ in the test dataset.

### Estimation of reporting bias in simulation of NHANES populations

**Figure 2:**
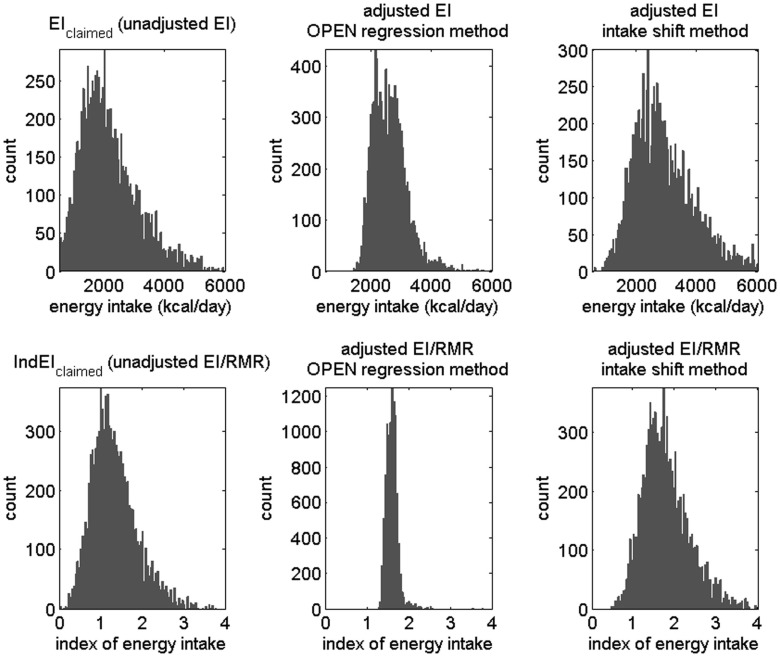
**Results from a simulation of non-Blacks from National Health and Nutrition Examination Survey (NHANES) 2007–2008 data**. Top row: histogram of energy intake (EI); bottom row: histogram of index of energy intake, i.e., energy intake divided by resting metabolic rate (RMR). Left column: unadjusted data, middle column: adjusted using the regression method (values outside bounds not shown for better clarity), and right column: adjusted using the intake shift method.

Prior to correction of dietary reporting, a simulation of 10,000 individuals predicted a baseline failure rate (i.e., IndEI_claimed_ out of range) of 31.9% in NHANES I and 32.5% in NHANES 2007–2008. Following the incorporation of the regression or intake shift models, the same simulations demonstrated substantial improvement over the unadjusted results, with diminished failure rates (Table 3; Figure 2).

**Table 3:** **Failure rate (percent outside of defined IndEI^a^ range after adjustment) of the regression and the intake shift models applied to NHANES I and NHANES 2007–2008 datasets for all adults, non-Blacks only, and non-Blacks age 40–69**.

Dataset	Regression (%)	Intake shift (%)
NHANES I all	0.67	5.95
NHANES I non-Blacks	0.70	5.64
NHANES I non-Blacks age 40–69^b^	0.53	4.06
NHANES 2007-2008 all	1.48	8.87
NHANES 2007-2008 non-Blacks	1.59	8.14
NHANES 2007-2008 non-Blacks age 40–69^b^	0.96	6.75

*^a^IndEI, index of energy intake = energy intake/resting metabolic rate; NHANES, National Health and Nutrition Examination Survey. Some small variation exists between the datasets for each method. All results show a much lower failure rate than the >33% in the raw data*.

*^b^Ages 40–69 represent the ages of participants in the training dataset observing protein and energy nutrition (OPEN)*.

**Figure 3:**
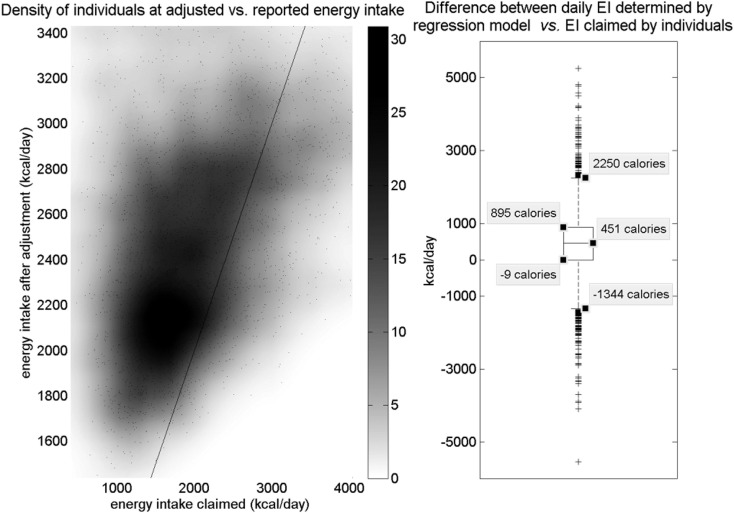
**Left: density of individuals according to adjusted EI vs. initial EI_claimed_ (energy intake claimed)**. Points represent data; darker regions indicate higher density of data points in that region. A line indicates unity. Most of the dense region lies above the line, indicating that most dietary intake was under-reported and shifted upward. A much less dense region below the line represents over-reporters. The majority of individuals claimed an intake in the range of 1,000–2,000 calories, and most of this group has been shifted to a range of about 1,900–2,400 calories. Another smaller group claimed between 2,000 and 2,500 calories and was shifted to around 2,700 calories. Dataset used includes only non-Blacks from National Health and Nutrition Examination Survey (NHANES) 2007–2008. Right: boxplot of calorie difference applied to individuals in the regression model. The interquartile range shows that most individuals under-reported and had EI (energy intake) adjusted upward. Some individuals still over-reported and had EI adjusted downward; however, the asymmetry of the box plot shows that many more individuals under-reported than over-reported. Outliers exist in both directions.

The regression method showed a failure rate of <2%. After adjusting, few individuals (e.g., in one test run, 23 of 10,000) are in the range of 0 < IndEI < 1, where individuals more often under-report in the crude, unadjusted data (Figure 2). Most misreporting was under-reporting (Figure 3, left). Using the OPEN regression model, the 25th and 75th percentiles for adjusting for misreporting were −9 and 895 calories. Although the great majority of the population was adjusted due to under-reporting, there were also some individuals whose EI was adjusted in a negative direction, indicating a small amount of over-reporting bias that occurs (Figure 3).

The intake shift method, with a failure rate of 4–9%, performed less well than the regression method but still showed a significant improvement over the unadjusted data of failure rate 32–33% (Figure 2). Using the intake shift model, we found that, on average, adding 905 calories to men’s intake and 600 calories to women’s intake best fit the population data in year 2007 for non-Blacks. For the 1971 population data, the best fit came from adding 758 calories to men’s intake and 613 calories to women’s intake. For both genders in both NHANES datasets, the caloric shift applied represented approximately 1/4 of the final EI for the gender group within that dataset.

The sensitivity analysis (Table 3) showed that while the best results occur with the age group corresponding to that of the OPEN study, the failure rates are nevertheless similar for all three groups in both cohorts.

## Discussion

In this study, we developed a new method to correct for EI under-reporting bias in a population, yielding a more plausible population distribution of total calories consumed daily. We created two models with different basic assumptions, and both greatly decrease the number of individuals in a test dataset whose claimed daily dietary intake is out of a physically possible range.

The variables in the regression on OPEN data showed a coefficient direction as expected. Intuitively, those with a low EI_claimed_ or IndEI_claimed_ are more likely to have under-reported by a greater amount. Our finding of increased under-reporting with weight agrees with previous findings that under-reporting increases with BMI (1). This correlation could arise from fear of stigma. It could also reflect the difficulty of recalling more or greater amounts of food for those with a higher weight who require a higher caloric intake. In the OPEN data, extent of under-reporting increases with EE, again suggesting that greater amounts of food intake are more difficult to recall (19).

The regression method reduces variance in the EI_claimed_ and IndEI_claimed_ ranges. The results appear close to the mean values for PAL from a tabulation of average PAL values from doubly labeled water studies (25).

The strong performance of the regression model suggests that the reasons for under-reporting may be fairly consistent among individuals, and that we are rather predictable in our reaction to a standardized self-reporting dietary recall.

The intake shift method may have performed less well than the regression method because it accounts only for average offset and not for individual variation. However, it still provides valuable insight in the case where the test dataset is believed to have participants with an EE distribution similar to that of participants in the training dataset.

Instead of adjusting, we could simply discard data that does not conform to normal food intake as determined by known PAL values from experimental data. However, this would require disgarding between a third and a half of the data, depending on the PAL value chosen. In addition, applying a single cut-off gives no provision for those who under-reported significantly yet were just above the cut-off, e.g., if a subject with a true IndEI of 1.85 instead recalled only enough for an IndEI_claimed_ of 1.4. The exclusion of only that data, which falls below a particular cut-off introduces bias.

The analysis of race showed a difference in under-reporting patterns, in particular that of Blacks, whose under-reporting does not seem to be influenced by factors that influence those of other racial/ethnic groups. With only 28 Black participants in OPEN, it is difficult to draw conclusions about these data. With a larger dataset of other racial/ethnic groups, it may be possible to make similar models for these groups.

### Limitations

The methods we have developed are meant to correct for total EI dietary recall bias in a population.

They cannot be used to accurately predict dietary intake for a particular individual on a particular date because day-to-day variations in individuals’ intake are averaged out over the population. For any individual in steady state over a prolonged period of constant weight, it is expected that dietary intake and EE should balance perfectly. This balance is not perfect over the short term such as the time of the OPEN study. While a longer study would improve this balance, it would also, as Subar mentions in the OPEN study paper, introduce more error from daily fluctuation in EI (19). In large populations such as NHANES/OPEN, fluctuations in the balance of intake and expenditure average out, making the error unimportant in overall interpretation of the model.

The methods were derived under the assumption of energy homeostasis, that is, EI equals EE, so that no weight change occurs. If the assumption did not hold, EE could not be assumed equal to EI. For example, EI of a participant losing weight would be lower than EE. Because OPEN exclusions included diets to alter weight, and because we excluded NHANES subjects who had reported being on a special diet or not eating typically that day, our study remains valid. However, it cannot be applied to populations whose subjects are altering their body weight during the study.

The training data set included individuals of age 40–69, but the test dataset included all adults in the NHANES data. A training dataset with a wider age range may be able to better characterize the variation in reporting with age. Still, our results show that while the models perform at a slightly lower failure rate in a population age 40–69, the results are in a similar range as those of a population of all adults.

## Conclusion

Under-reporting can be a serious problem in dietary studies. We have presented two methods for adjusting for under-reporting, and both showed a substantial improvement in biological plausibility over the raw data. These methods can better inform quantitative nutritional research in populations.

In addition to providing a tool for other studies of diet in populations, these methods may be useful in predicting under-reporting for many other self-reported habits, such as smoking or alcohol consumption. Correcting for these would require an experimental dataset with biomarkers of actual consumption, in addition to a self-reported collection from the participants.

## Author Contributions

Joanna Lankester designed the research; Joanna Lankester conducted the research; Joanna Lankester, Sharon Perry, and Julie Parsonnet analyzed and interpreted data; Joanna Lankester drafted paper; and Joanna Lankester, Sharon Perry, and Julie Parsonnet made substantial revisions to the paper. All authors read and approved the final manuscript.

## Conflict of Interest Statement

The authors declare that the research was conducted in the absence of any commercial or financial relationships that could be construed as a potential conflict of interest.

## Abbreviations

BMI, body mass index; EE, energy expenditure; EI, energy intake (actual, not claimed); EI_claimed_, energy intake claimed in dietary recall (in calories); IndEI, index of energy intake EI/RMR; IndEI_claimed_, EI_claimed_/RMR; NHANES, National Health and Nutrition Examination Survey; OPEN, observing protein and energy nutrition; PAL, physical activity level energy expenditure divided by resting metabolic rate; RMR, calculated resting metabolic rate.
